# Correction: An analysis of the effects of sharing research data, code, and preprints on citations

**DOI:** 10.1371/journal.pone.0315776

**Published:** 2024-12-10

**Authors:** The PLOS ONE Staff

## Notice of Republication

This article was republished on November 22, 2024, to correct an error in the competing interests statement. The publisher apologises for the error. Please download this article again to view the correct version.
