# Supplementary material for: An analysis of the effects of sharing research data, code, and preprints on citations
Source: PLoS One. 2024 Oct 30;19(10):e0311493. doi: 10.1371/journal.pone.0311493 (PMC11524460; doi:10.1371/journal.pone.0311493)
Supplement: S1 Appendix — (PDF) [file pone.0311493.s001.pdf]

Supporting Information

We show in this Appendix results for a few more models in order to further confirm our results. Firstly, a base model adding code generated as a variable shows a small yet significant negative effect related to it (Table 7). This effect goes away when controlling for disciplines, therefore we consider it spurious. When considering OSI interactions (Table 8), we find a further negative effect provided by code generated and code shared. This surprising result may be an artifact of the dataset, that we are unsure how to explain. Next, we show how different preprint servers are associated with varying degrees of citation impact (Table 9). Lastly, we check a full model using as dependent variables the citation counts up to 1 year after publication (Table 10). We still find the same results as using the full citation counts, albeit with a smaller magnitude as expected.

Table 7. Results for the base model with code generated OSI.

	Dependent variable:	
	n_cit_tot_log	
	OLS	*robust linear*
	(1)	(2)
n_authors_log	0.266*** (0.005)	0.255*** (0.004)
n_references_tot_log	0.195*** (0.005)	0.200*** (0.005)
p_year	−0.357*** (0.001)	−0.370*** (0.001)
p_month	−0.037*** (0.001)	−0.039*** (0.001)
h_index_mean_log	0.141*** (0.003)	0.142*** (0.003)
C(is_plos)True	0.095*** (0.009)	0.106*** (0.008)
C(is_plos_one)True	−0.348*** (0.007)	−0.352*** (0.007)
C(Data_Shared)True	−0.005 (0.034)	−0.007 (0.033)
C(Data_Location)Online	−0.003 (0.034)	−0.003 (0.034)
C(Data_Location)Supplementary Information	0.020 (0.034)	0.024 (0.033)
C(Repositories_data_bool)True	0.041*** (0.008)	0.040*** (0.008)
C(Code_Generated)True	−0.022*** (0.005)	−0.017*** (0.005)
C(Code_Shared)True	0.070 (0.107)	0.075 (0.104)
C(Code_Location)Online	−0.111	−0.132

	(0.107)	(0.105)
C(Code_Location)Supplementary Information	−0.137	−0.138
	(0.108)	(0.105)
C(Preprint_Match)True	0.188***	0.160***
	(0.006)	(0.006)
Constant	720.947***	746.881***
	(2.705)	(2.638)
Observations	121,999	121,999
R^2	0.409	
Adjusted R^2	0.408	
Residual Std. Error (df = 121982)	0.775	0.723
F Statistic	5,266.481*** (df = 16; 121982)	
Note:		*p<0.1; **p<0.05; ***p<0.01

Table 8. Results for the base model with interactions among OSI.

	Dependent variable:	
	n_cit_tot_log	
	OLS	*robust linear*
	(1)	(2)
n_authors_log	0.266***	0.254***
	(0.005)	(0.004)
n_references_tot_log	0.195***	0.200***
	(0.005)	(0.005)
p_year	−0.357***	−0.370***
	(0.001)	(0.001)
p_month	−0.037***	−0.039***
	(0.001)	(0.001)
h_index_mean_log	0.141***	0.142***
	(0.003)	(0.003)
C(is_plos)True	0.096***	0.107***
	(0.009)	(0.008)
C(is_plos_one)True	−0.350***	−0.352***
	(0.007)	(0.007)
C(Data_Shared)True	−0.007	−0.009
	(0.034)	(0.033)
C(Data_Location)Online	−0.003	−0.003
	(0.034)	(0.034)
C(Data_Location)Supplementary Information	0.022	0.025
	(0.034)	(0.033)
C(Repositories_data_bool)True	0.044***	0.041***
	(0.009)	(0.009)
C(Preprint_Match)True	0.190***	0.160***
	(0.007)	(0.007)
C(Code_Generated)True	−0.015***	−0.010*

	(0.005)	(0.005)
C(Code_Shared)True	0.144	0.147
	(0.108)	(0.105)
C(Code_Location)Online	−0.120	−0.142
	(0.107)	(0.105)
C(Code_Location)Supplementary Information	−0.166	−0.166
	(0.108)	(0.105)
C(Repositories_data_bool)True:C(Preprint_Match)True	−0.007	0.002
	(0.012)	(0.012)
C(Code_Generated)True:C(Code_Shared)True	−0.079***	−0.077***
	(0.017)	(0.017)
Constant	720.952***	746.876***
	(2.705)	(2.638)
Observations	121,999	121,999
R^2	0.409	
Adjusted R^2	0.409	
Residual Std. Error (df = 121980)	0.775	0.723
F Statistic	4,683.261*** (df = 18; 121980)	
Note:		*p<0.1; **p<0.05; ***p<0.01

Table 9. Results for the base model with preprint servers (considering only those mentioned in 500 or more publications part of the dataset).

	Dependent variable:	
	n_cit_tot_log	
	OLS	*robust linear*
	(1)	(2)
n_authors_log	0.259***	0.250***
	(0.005)	(0.004)
n_references_tot_log	0.201***	0.204***
	(0.005)	(0.005)
p_year	−0.360***	−0.372***
	(0.001)	(0.001)
p_month	−0.037***	−0.039***
	(0.001)	(0.001)
h_index_mean_log	0.143***	0.143***
	(0.003)	(0.003)
C(is_plos)True	0.094***	0.106***
	(0.009)	(0.009)
C(is_plos_one)True	−0.344***	−0.346***
	(0.007)	(0.007)
C(Data_Shared)True	−0.010	−0.013
	(0.034)	(0.033)
C(Data_Location)Online	−0.001	0.002
	(0.034)	(0.034)

C(Data_Location)Supplementary Information	0.027<br>(0.034)	0.030<br>(0.033)
C(Repositories_data_bool)True	0.043***<br>(0.009)	0.041***<br>(0.008)
C(Code_Shared)True	0.060<br>(0.107)	0.059<br>(0.105)
C(Code_Location)Online	−0.107<br>(0.108)	−0.119<br>(0.105)
C(Code_Location)Supplementary Information	−0.131<br>(0.108)	−0.125<br>(0.106)
C(Preprint_Match)True	0.689**<br>(0.345)	0.264<br>(0.338)
C(Preprint_Server)bioRxiv	0.189***<br>(0.027)	0.191***<br>(0.027)
C(Preprint_Server)Journal of Medical Internet Research	0.517***<br>(0.038)	0.480***<br>(0.037)
C(Preprint_Server)medRxiv	0.470***<br>(0.030)	0.381***<br>(0.029)
C(Preprint_Server)N/A	0.721**<br>(0.346)	0.309<br>(0.339)
C(Preprint_Server)Protocols.io	−0.047<br>(0.043)	−0.040<br>(0.042)
C(Preprint_Server)PsyArXiv	0.181***<br>(0.039)	0.139***<br>(0.038)
C(Preprint_Server)Research Square	0.191***<br>(0.029)	0.194***<br>(0.028)
Constant	726.760***<br>(2.754)	751.681***<br>(2.695)
Observations	120,195	120,195
R^2	0.413	
Adjusted R^2	0.413	
Residual Std. Error (df = 120172)	0.772	0.721
F Statistic	3,838.518*** (df = 22; 120172)	
Note:		*p<0.1; **p<0.05; ***p<0.01

Table 10. Results for the full model with dependent variable as citation data for 1 year from publication.

	Dependent variable:	
	n_cit_1_log	
	OLS	*robust linear*
	(1)	(2)
n_authors_log	0.131***<br>(0.004)	0.109***<br>(0.003)
n_references_tot_log	0.113***<br>(0.004)	0.117***<br>(0.004)

p_year	0.020***	0.015***
	(0.001)	(0.001)
p_month	−0.013***	−0.013***
	(0.001)	(0.001)
h_index_mean_log	0.052***	0.050***
	(0.003)	(0.002)
C(is_plos)True	0.129***	0.149***
	(0.007)	(0.006)
C(is_plos_one)True	−0.303***	−0.302***
	(0.006)	(0.005)
C(Data_Shared)True	0.015	0.015
	(0.025)	(0.023)
C(Data_Location)Online	0.007	0.008
	(0.025)	(0.023)
C(Data_Location)Supplementary Information	0.003	0.005
	(0.025)	(0.023)
C(Repositories_data_bool)True	0.012*	0.011*
	(0.006)	(0.006)
C(Code_Shared)True	0.084	0.075
	(0.081)	(0.075)
C(Code_Location)Online	−0.091	−0.092
	(0.081)	(0.075)
C(Code_Location)Supplementary Information	−0.101	−0.087
	(0.082)	(0.075)
C(Preprint_Match)True	0.133***	0.099***
	(0.004)	(0.004)
C(division_1)True	0.047***	0.049***
	(0.004)	(0.004)
C(division_2)True	0.019***	0.029***
	(0.005)	(0.004)
C(division_3)True	0.003	−0.001
	(0.005)	(0.004)
C(division_4)True	−0.015**	−0.007
	(0.007)	(0.006)
C(division_5)True	−0.037***	−0.030***
	(0.008)	(0.007)
C(division_6)True	−0.041***	−0.044***
	(0.008)	(0.007)
C(division_7)True	0.006	0.0003
	(0.009)	(0.008)
C(division_8)True	−0.073***	−0.054***
	(0.009)	(0.008)
C(division_9)True	−0.119***	−0.104***
	(0.009)	(0.008)

C(division_10)True	0.045***	0.056***
	(0.010)	(0.009)
C(division_11)True	−0.026**	−0.019*
	(0.012)	(0.011)
C(division_12)True	−0.056***	−0.057***
	(0.012)	(0.011)
C(division_13)True	−0.037**	−0.030**
	(0.014)	(0.013)
C(division_14)True	−0.072***	−0.062***
	(0.016)	(0.015)
C(division_15)True	−0.024	−0.012
	(0.018)	(0.017)
C(division_16)True	0.010	0.0002
	(0.019)	(0.018)
C(division_17)True	−0.056***	−0.045**
	(0.020)	(0.018)
C(division_18)True	−0.032***	−0.033***
	(0.011)	(0.010)
Constant	−40.101***	−29.688***
	(2.385)	(2.198)
Observations	106,733	106,733
R^2	0.120	
Adjusted R^2	0.120	
Residual Std. Error (df = 106699)	0.542	0.522
F Statistic	442.350*** (df = 33; 106699)	
Note:		*p<0.1; **p<0.05; ***p<0.01
